# Fear-driven predator–prey dynamics with prey refuge: analytical framework and physics-informed neural network approach

**DOI:** 10.3389/frai.2026.1868693

**Published:** 2026-08-10

**Authors:** G. Ramraj, T. Poornima

**Affiliations:** Department of Mathematics, Vellore Institute of Technology, Vellore, India

**Keywords:** bifurcation, fear effect, physics-informed neural networks, predator–prey model, prey refuge, Routh–Hurwitz criterion, stability analysis

## Abstract

Ecological communities are governed not only by direct consumption but also by indirect behavioral responses triggered by perceived predation risk. Predator-induced fear substantially suppresses prey reproductive output and foraging efficiency even when lethal predation is absent, a mechanism documented across a wide range of taxa including songbirds, ungulates, and marine invertebrates. Motivated by this observation, we formulate a deterministic two-species model that simultaneously incorporates fear-mediated prey growth reduction, partial prey refuge, density-dependent intraspecific regulation, and predator self-interference. The proposed model is distinguished from existing fear–refuge frameworks by jointly embedding four ecological mechanisms within a single functional-response denominator 1 + *kv* + *αu*, producing qualitatively novel stability thresholds absent in models incorporating only subsets of these effects. Biological admissibility is rigorously established through positivity and uniform boundedness proofs. The boundedness condition $c\beta \left(1-\delta \right)<2\sqrt{a\eta }$ is derived from first principles by applying Sylvester's criterion to the cross-interaction quadratic form. Three ecologically meaningful equilibria are identified and their local stability is characterized via carefully re-derived Jacobian linearization and the Routh–Hurwitz criterion. Numerical experiments via the fourth-order Runge–Kutta method reveal convergence to a stable coexistence equilibrium across the explored parameter ranges, with the approach transitioning from a stable node to a stable focus as predation intensifies; no sustained oscillations are observed. A physics-informed neural network (PINN) is constructed with four hidden layers of 64 neurons each, tanh activations, Adam followed by L-BFGS training over 10,000 iterations, and 200 collocation points, achieving maximum absolute errors of 7.98 × 10^−3^ (prey) and 5.83 × 10^−3^ (predator) relative to the RK4 reference. Comparison with a data-driven neural network of identical architecture shows a fivefold accuracy improvement from the physics-informed loss. Numerical evidence for global stability is reported; rigorous Lyapunov-based analysis is identified as future work.

## 1 Introduction

Predator–prey interactions occupy a central position in mathematical ecology because they provide tractable yet rich models for studying how species regulate one another. The pioneering frameworks of Lotka (1925) and Volterra (1926) demonstrated that purely consumptive encounters generate intrinsic oscillatory cycles. However, these classical formulations neglect several ecologically important mechanisms.

Extensive evidence has established that predators influence prey through two pathways (Preisser et al., 2005): (i) direct consumptive removal and (ii) indirect non-consumptive behavioral modification (Lima, 1998; Zanette et al., 2011). Fear-driven modifications include heightened vigilance, reduced foraging, and lowered reproductive investment (Cresswell, 2011). Wang et al. (2016) formalized this by introducing a multiplicative fear function 1/(1 + *kv*) into the prey growth term, showing that *k* can stabilize or destabilize coexistence. Subsequent studies incorporated seasonal variation (Pal et al., 2019), age structure (Liu and Jiang, 2019), and Allee effects (Sasmal, 2018).

A second mechanism is spatial prey refuge, where a fraction of prey occupies inaccessible microhabitats (McNair, 1986; Pal et al., 2018). Refuge typically broadens the parameter region supporting stable coexistence and can eliminate limit cycles (Kar, 2005; Gonz'alez-Olivares and Ramos-Jiliberto, 2003). The combined effect of fear and refuge was explored by Kumar and Dubey (2019), who found that these mechanisms interact non-trivially.

The role of fractional-order dynamics in predator–prey modeling has received growing attention, as it captures memory effects and hereditary properties. Fractional prey–predator models with fear effects and refuge (Das and Samanta, 2021) reveal that the order of differentiation significantly alters stability thresholds. Fractional Leslie–Gower models with modified Holling-type responses and fear (Yousef et al., 2022) show that non-integer dynamics enrich the bifurcation landscape. Harvesting in fractional predator–prey systems () further complicates the stability picture. Memory-based transmission modeling using the Caputo derivative (Chauhan, 2025) illustrates how fractional calculus captures realistic biological dynamics that integer-order systems overlook. These advances motivate the present study, which focuses on the integer-order setting as a necessary first step before fractional extensions.

Predator self-interference captures intraspecific competition among predators (Hassell, 1978), preventing unbounded growth, and ensuring admissibility (Beddington, 1975).

Despite these individual advances, models simultaneously incorporating predator-induced fear, proportional prey refuge, prey crowding, and predator self-interference within a single unified framework remain scarce. The present work is specifically distinguished from the existing literature as follows. The model of Wang et al. (2016) includes only fear (*k*), with no refuge (*δ* = 0), no prey crowding (*α* = 0), and no predator self-interference (*η* = 0). Kumar and Dubey (2019) combined fear and refuge but excluded prey crowding and predator interference. The present model extends both by jointly embedding all four mechanisms in the combined fear–crowding denominator 1 + *kv* + *αu*. This coupling makes the effective prey growth rate a decreasing function of *both* population densities simultaneously, producing cross-inhibition feedback loops and novel stability thresholds absent in prior models. The term *ηv*^2^ prevents unbounded predator growth without requiring a Beddington–DeAngelis denominator. Furthermore, the integration of PINNs as a validation tool for this class of ecological ODE has not been systematically explored in the literature.

Physics-informed neural networks (PINNs) (Raissi et al., 2019) embed governing equations directly into the loss function, requiring no large training datasets (Karniadakis et al., 2021). Their mesh-free nature suits complex dynamical systems (Lenz, 2023; Sun et al., 2020), with applications in epidemic modeling (Cai et al., 2021) and pharmacokinetics (Yazdani et al., 2020).

### 1.1 Motivation and objectives

Predator–prey systems with fear, refuge, and nonlinear functional responses can exhibit rich dynamical structures, including multiple equilibria and, in some parameter regimes, bifurcations to oscillatory dynamics. Accurately simulating such systems is challenging near these transitions. Physics-informed neural networks offer a complementary computational framework that enforces biological constraints while providing smooth approximations, with natural extensions to inverse problems and parameter estimation.

The main objectives are:

- To formulate a biologically realistic model incorporating fear, refuge, density-dependent prey growth, and predator interference, and to clearly differentiate this model from the existing literature.
- To establish well-posedness by proving positivity and rigorously deriving uniform boundedness.
- To determine all feasible equilibria and analyze local stability using carefully derived Jacobian linearization.
- To analyze coexistence stability and the sensitivity of the equilibrium to the key ecological parameters.
- To implement a PINN with full architectural and training specification and validate against RK4 and a data-driven neural network baseline.
- To provide an eight-item future research roadmap.

## 2 Model formulation

Let *u*(*t*) and *v*(*t*) denote prey and predator densities. The proposed system is


$$\begin{array}{l} \begin{array}{cc} \frac{du}{dt} & =\frac{r\;u}{1+kv+\alpha u}-au^{2}-\beta \left(1-\delta \right)uv,\\ \frac{dv}{dt} & =c\beta \left(1-\delta \right)uv-mv-\eta v^{2}, \end{array} \end{array}$$ (1)

with *u*(0) > 0, *v*(0) > 0; all parameters strictly positive, *δ* ∈ [0, 1), *c* ∈ (0, 1] (Table 1).

**Table 1:** Model parameters, biological interpretations, and units.

Param.	Meaning	Units	Biological interpretation
*r*	Prey growth rate	time^−1^	Intrinsic per-capita birth rate in absence of predators.
*k*	Fear coefficient	predator^−1^	Per-predator reduction in prey birth rate.
α	Prey crowding	prey^−1^	Density-dependent reduction in prey birth rate.
*a*	Prey self-competition	(prey·time)^−1^	Quadratic mortality at high prey density.
β	Predation rate	(predator·time)^−1^	Rate of successful predation on exposed prey.
*δ*	Refuge fraction	dimensionless	Proportion of prey protected (*δ* = 0: none; → 1: full).
*c*	Conversion efficiency	dimensionless	Prey biomass converted to predator reproduction.
*m*	Predator mortality	time^−1^	Natural per-capita death rate of predators.
η	Predator self-competition	(predator·time)^−1^	Density-dependent predator mortality.

The prey equation models growth in which the effective per-capita birth rate *r*/(1 + *kv* + *αu*) is reduced by predator-induced fear (through *kv*) and by intraspecific crowding (through *αu*). The term −*au*^2^ represents additional density-dependent mortality. Predation acts only on the exposed fraction (1 − *δ*) of prey. The predator equation models biomass conversion at rate *cβ*(1 − *δ*), natural mortality *m*, and intraspecific competition *η* that prevents unbounded growth.

### 2.1 Comparison with related models

Wang et al. (2016): fear only (*k* > 0), *δ* = *α* = *η* = 0, Lotka–Volterra predation. Kumar and Dubey (2019): fear + refuge (*δ* > 0), but *α* = *η* = 0. Present model: all four mechanisms simultaneously. The denominator 1 + *kv* + *αu* is the key structural novelty: both species densities enter the functional response, creating coupled nonlinear feedbacks that shift nullcline geometry, alter equilibrium positions, and modify Routh–Hurwitz conditions in ways that cannot be recovered by superposing single-mechanism models.

## 3 Well-posedness: positivity and boundedness

### 3.1 Positive invariance

** Theorem 1.** *Let* (*u*(*t*), *v*(*t*)) *be any solution of Equation 1 with positive initial conditions* *u*(0) > 0 *and* *v*(0) > 0. *Then* *u*(*t*) > 0 *and* *v*(*t*) > 0 *for all* *t* > 0.

*Proof:* Write the first equation as $\overset{•}{u}=u\left(t\right)\;F\left(t\right)$ where *F*(*t*) = *r*/(1+*kv*+*αu*)−*au*−β(1−*δ*)*v*. Since the denominator 1+*kv*+*αu* ≥ 1 > 0 for all *u, v* ≥ 0, *F* is continuous on any finite interval. Integration gives $u(t)=u(0)\;\exp\text{​}\left(\int_{0}^{t}F(s)\;ds\right)>0$, since *u*(0) > 0 and the exponential is strictly positive. An identical argument applies to the predator: $v(t)=v(0)\;\exp\text{​}\left(\int_{0}^{t}G(s)\;ds\right)>0$ where *G*(*t*) = *cβ*(1 − *δ*)*u* − *m* − *ηv*. Hence ${\mathbb{R}}_{+}^{2}=\left\{u>0,v>0\right\}$ is positively invariant.

### 3.2 Uniform boundedness

** Theorem 2.** *Suppose* $c\beta \left(1-\delta \right)<2\sqrt{a\eta }$. *Then all solutions are uniformly ultimately bounded and enter*


$$\Omega =\text{​}\left\{(u,v)\in {\mathbb{R}}_{+}^{2}\;|\;u+v\leq \frac{{\left(r+M\right)}^{2}}{4Ma}+\varepsilon \right\},\;M=\min\{m,1\}.$$

*Proof:* Define *h*(*t*) = *u*(*t*) + *v*(*t*). Differentiating along solutions and using 1 + *kv* + *αu* ≥ 1 and (1 − *c*)*β*(1 − *δ*) ≥ 0 Equation 2,


$$\begin{array}{l} \frac{dh}{dt}\leq ru-au^{2}-mv-\eta v^{2}-\left(1-c\right)\beta \left(1-\delta \right)uv. \end{array}$$ (2)

Adding *Mh* to both sides and using *M* ≤ *m*,


$$\frac{dh}{dt}+Mh\leq -au^{2}+\left(r+M\right)u-\eta v^{2}-\left(1-c\right)\beta \left(1-\delta \right)uv.$$

The right-hand side equals (*r* + *M*)*u* − *Q*(*u, v*) where *Q*(*u, v*) = *au*^2^ + *ηv*^2^ + (1 − *c*)β(1 − *δ*)*uv*. This quadratic form is associated with the matrix


$$A=\left(\begin{array}{cc} a & \frac{\left(1-c\right)\beta \left(1-\delta \right)}{2}\\ \frac{\left(1-c\right)\beta \left(1-\delta \right)}{2} & \eta \end{array}\right).$$

By Sylvester's criterion, *A* is positive definite iff *a* > 0 (satisfied) and


$$\det\left(A\right)=a\eta -\frac{{\left[\left(1-c\right)\beta \left(1-\delta \right)\right]}^{2}}{4}>0\Leftrightarrow \left(1-c\right)\beta \left(1-\delta \right)<2\sqrt{a\eta }.$$

Since *c* ∈ (0, 1], the hypothesis $c\beta \left(1-\delta \right)<2\sqrt{a\eta }$ implies $\left(1-c\right)\beta \left(1-\delta \right)\leq c\beta \left(1-\delta \right)<2\sqrt{a\eta }$, so *A* is positive definite and *Q*(*u, v*) ≥ 0. Therefore ḣ + *Mh* ≤ −*au*^2^ + (*r* + *M*)*u* ≤ (*r* + *M*)^2^/(4*a*) = :*K*. The comparison lemma gives ${\mathrm{limsup}}_{t\to \infty }h\left(t\right)\leq K/M={\left(r+M\right)}^{2}/\left(4Ma\right)$, establishing uniform ultimate boundedness.

** Remark 1.** *The condition* $c\beta \left(1-\delta \right)<2\sqrt{a\eta }$ *constrains the product of conversion efficiency, predation rate, and exposed-prey fraction relative to the geometric mean of the two intraspecific competition coefficients, ensuring cross-species energy transfer cannot overcome combined self-limitation. For the baseline parameters of Section 6, this evaluates to* 0.336 < 0.632 *and is comfortably satisfied*.

## 4 Equilibrium analysis

### 4.1 Equilibrium points

**(i) Trivial equilibrium** ***E*_0_(0, 0):** Always exists.

**(ii) Prey-only equilibrium** ***E*_1_ (*u*_1_, 0):** Setting *v* = 0 in $\overset{•}{u}=0$ gives *aαu*^2^ + *au* − *r* = 0, with unique positive root in Table 2


$$u_{1}=\frac{-1+\sqrt{1+4\alpha r/a}}{2\alpha },$$

which reduces to *r*/*a* when *α* = 0.

**Table 2:** Equilibria, existence conditions, and stability.

Equilibrium	Existence	Local stability
*E*_0_(0, 0)	Always	Saddle (always unstable)
*E*_1_(*u*_1_, 0)	Always; $u_{1}=\left(-1+\sqrt{1+4\alpha r/a}\right)/\left(2\alpha \right)$	L.A.S. iff *cβ*(1 − *δ*)*u*_1_ < *m*
E^*^(u^*^, v^*^)	Cond. Equation 5	L.A.S. iff (Equation 7)

**(iii) Interior equilibrium** E^*^(u^*^, v^*^): Both components satisfy


$$\begin{array}{ll} \frac{r}{1+kv^{*}+\alpha u^{*}}-au^{*}-\beta \left(1-\delta \right)v^{*} & =0, \end{array}$$ (3)

$$\begin{array}{ll} c\beta \left(1-\delta \right)u^{*}-m-\eta v^{*} & =0. \end{array}$$ (4)

From Equations 3,4: v^*^ = (*cβ*(1 − *δ*)u^*^ − *m*)/*η* > 0 requires $u^{*}>u_{\min}:=m/\left(c\beta \left(1-\delta \right)\right)$. Interior equilibrium exists when


$$\begin{array}{l} r>\frac{am}{c\beta \left(1-\delta \right)}\left(1+\frac{\alpha m}{c\beta \left(1-\delta \right)}\right). \end{array}$$ (5)

### 4.2 Jacobian matrix

For a general point (*u, v*), define Φ = 1 + *kv* + *αu*. Partial differentiation of Equation 1 gives:


$$\begin{array}{ll} J_{11} & =\frac{r}{\Phi }-\frac{r\alpha u}{\Phi^{2}}-2au-\beta \left(1-\delta \right)v=\frac{r\left(1\;+\;kv\right)}{\Phi^{2}}\\ -2au-\beta \left(1-\delta \right)v,\\ J_{12} & =-\frac{rku}{\Phi^{2}}-\beta \left(1-\delta \right)u,\;J_{21}=c\beta \left(1-\delta \right)v,\\ J_{22} & =c\beta \left(1-\delta \right)u-m-2\eta v. \end{array}$$

### 4.3 Stability of *E*_0_(0, 0)

At (0, 0), *J* = diag(*r*, −*m*). Eigenvalues λ_1_ = *r* > 0, λ_2_ = −*m* < 0: saddle, always unstable. Biologically, total extinction is never an attractor; prey can always invade from low density.

### 4.4 Stability of *E*_1_(*u*_1_, 0)

*J*(*E*_1_) is upper triangular with eigenvalues λ_1_ = *J*_11_ and λ_2_ = *cβ*(1 − *δ*)*u*_1_ − *m*. Using the equilibrium condition *r*/Φ_1_ = *au*_1_ (where Φ_1_ = 1 + *αu*_1_):


$$J_{11}=\frac{r}{\Phi_{1}}-\frac{r\alpha u_{1}}{\Phi_{1}^{2}}-2au_{1}=\frac{au_{1}}{\Phi_{1}}-2au_{1}=au_{1}\left(\frac{1}{\Phi_{1}}-2\right)<0,$$

since Φ_1_ ≥ 1 implies 1/Φ_1_ ≤ 1 < 2. *E*_1_ is L.A.S. iff


$$\begin{array}{l} c\beta \left(1-\delta \right)u_{1}<m. \end{array}$$ (6)

Larger refuge (*δ* ↑) or smaller conversion efficiency (*c* ↓) tighten this condition and stabilize the predator-free state.

### 4.5 Stability of E^*^(u^*^, v^*^)

At E^*^, the equilibrium condition *cβ*(1 − *δ*)u^*^ = *m* + ηv^*^ simplifies *J*_22_:


$$J_{22}^{*}=c\beta \left(1-\delta \right)u^{*}-m-2\eta v^{*}=\eta v^{*}-2\eta v^{*}=-\eta v^{*}<0.$$

The remaining entries are:


$$\begin{array}{lll} J_{11}^{*} & = & \frac{r\left(1+kv^{*}\right)}{\Phi^{*2}}-2au^{*}-\beta \left(1-\delta \right)v^{*},\\ J_{12}^{*} & = & -\frac{rku^{*}}{\Phi^{*2}}-\beta \left(1-\delta \right)u^{*}<0,\\ J_{21}^{*} & = & c\beta \left(1-\delta \right)v^{*}>0. \end{array}$$

By the Routh–Hurwitz criterion, E^*^ is L.A.S. iff


$$\begin{array}{l} \text{tr}\left(J^{*}\right)=J_{11}^{*}+J_{22}^{*}<0\;\text{and }\det\left(J^{*}\right)=J_{11}^{*}J_{22}^{*}-J_{12}^{*}J_{21}^{*}>0. \end{array}$$ (7)

### 4.6 Summary of equilibria

Biological interpretation: (1) *E*_0_ is always a saddle; total extinction is never stable. (2) *E*_1_ is stable when predators cannot invade the prey-at-carrying-capacity state. (3) E^*^ represents stable long-term coexistence; its stability is promoted by strong self-limitation (*a*, *η*) and reduced by intense predation (*β*) or fear (*k*).

## 5 Physics-informed neural network implementation

Physics-informed neural networks embed governing differential equations directly into the training loss, enforcing biological laws at collocation points throughout the solution domain. All implementation details are specified below to ensure full reproducibility.

### 5.1 Network architecture

The PINN is a fully connected feedforward network:

- **Input layer:** 1 neuron—scalar time *t* ∈ [0, *T*].
- **Hidden layers:** 4 layers × 64 neurons.
- **Activation function:** tanh, elementwise after each hidden layer—selected for smoothness, bounded range (−1, 1), and suitability for smooth ODE solutions (Raissi et al., 2019).
- **Output layer:** 2 neurons (*û*, $\hat{v}$), linear (no activation).
- **Weight initialization:** Glorot uniform (Glorot and Bengio, 2010).
- **Total trainable parameters:** $\underset{12,480\;\text{weights}}{\underset{︸}{1\times 64+3\left(64\times 64\right)+64\times 2}}+\underset{258\;\text{biases}}{\underset{︸}{4\times 64+2}}=12,738$.

### 5.2 Collocation points and domain

*N* = 200 uniformly spaced collocation points on [0, *T*] with *T* = 50. Initial conditions are enforced separately.

### 5.3 Automatic differentiation

Time derivatives are computed by reverse-mode automatic differentiation (Baydin et al., 2018)—exact derivatives of the network's computational graph with respect to *t*, not finite-difference approximations.

### 5.4 PINN residuals


$$\begin{array}{ll} R_{u} & =\frac{dû}{dt}-\left[\frac{rû}{1+k\hat{v}+\alpha û}-a{û}^{2}-\beta \left(1-\delta \right)û\hat{v}\right], \end{array}$$ (R1)

$$\begin{array}{ll} R_{v} & =\frac{d\hat{v}}{dt}-\left[c\beta \left(1-\delta \right)û\hat{v}-m\hat{v}-\eta {\hat{v}}^{2}\right]. \end{array}$$ (R2)

### 5.5 Loss function

The total loss is


$$L\left(\theta \right)=L_{\text{ODE}}+\lambda L_{\text{IC}},\;L_{\text{ODE}}=\frac{1}{N}\overset{N}{\underset{i=1}{\sum }}\left[R_{u}{\left(t_{i}\right)}^{2}+R_{v}{\left(t_{i}\right)}^{2}\right],$$

where


$$L_{\text{IC}}={\left(û\left(0\right)-u_{0}\right)}^{2}+{\left(\hat{v}\left(0\right)-v_{0}\right)}^{2},$$

and λ = 10 up-weights the initial condition constraint.

### 5.6 Optimization strategy

Training uses two phases (Raissi et al., 2019):

1. **Adam:** 8,000 epochs, initial learning rate 10^−3^, step-decay factor 0.9 every 1, 000 epochs—facilitates rapid early convergence.
2. **L-BFGS-B:** up to 2,000 iterations with Wolfe line search (Liu and Nocedal, 1989)—achieves high-precision convergence using curvature information.

Total: 10,000 effective iterations.

### 5.7 Convergence criteria

Training terminates when: (i) total loss $L<10^{-6}$; (ii) relative change in loss between Adam epochs < 10^−8^; or (iii) L-BFGS gradient ℓ^∞^-norm < 10^−7^ (Table 3).

**Table 3:** Convergence to E^*^ from five initial conditions (baseline parameters, *t*_end_ = 200).

*u*(0)	*v*(0)	Final *u*	Final *v*	Outcome
0.5	0.1	1.4315	0.4050	Coexistence
2.0	0.5	1.4315	0.4050	Coexistence
3.0	0.1	1.4315	0.4050	Coexistence
0.2	0.05	1.4315	0.4050	Coexistence
4.0	0.8	1.4315	0.4050	Coexistence

### 5.8 Computational tradeoff: PINN vs. RK4

A natural question is whether the PINN is justified for a simple two-dimensional ODE. We address this directly. **RK4 advantages:** millisecond runtime, fourth-order convergence with provable error bounds, no hyperparameters; used here as the gold-standard reference (Table 4). **PINN justification:** (i) Produces a smooth closed-form approximation $N_{\theta }\left(t\right)$ evaluable at arbitrary *t* without re-integration. (ii) Solves inverse problems by differentiating through the loss; RK4 requires separate adjoint computations. (iii) Extends to fractional-order, PDE, and stochastic variants. (iv) Validates machine learning for enforcing biological constraints. The primary purpose here is *proof-of-concept*: demonstrating < 1% error to establish the PINN as a reliable platform for future, more complex extensions.

**Table 4:** Comparison of maximum absolute errors between the data-driven neural network (DDNN) and the physics-informed neural network (PINN) for prey and predator densities.

Method	Max error (prey)	Max error (predator)
DDNN (data-driven)	4.2 × 10^−2^	3.7 × 10^−2^
PINN (ours)	7.98 × 10^−3^	5.83 × 10^−3^
Improvement	≈ 5 ×	≈ 6 ×

### 5.9 Comparison with data-driven neural network

To quantify the benefit of physics-informed training, we compare the PINN against a purely data-driven neural network (DDNN) of identical architecture, no ODE constraints.

The PINN achieves a fivefold error reduction. Moreover, the DDNN extrapolates poorly beyond its training window, whereas the PINN maintains accuracy throughout [0, *T*] by construction.

For this comparison both networks share the identical architecture, initialization, and optimizer; the data-driven network is trained purely on supervised samples of the trajectory, with the ODE residual removed. Its larger error therefore reflects weaker generalization away from the supplied samples—both between sampled points and toward the end of the horizon—where the PINN's embedded ODE constraint continues to enforce the dynamics. We note, for completeness, that with sufficiently dense supervised data spanning the whole horizon a data-driven network of this size can attain comparable accuracy; the physics-informed formulation is thus most advantageous in the data-scarce and extrapolatory regime relevant here, where the governing equations supply the supervision that data alone do not.

### 5.10 Positioning relative to recent PINN advances

It is instructive to situate the present scheme within the rapidly evolving PINN literature. Pandey et al. (2026) recently proposed a Crank–Nicolson enhanced two-phase PINN for parabolic partial differential equations, demonstrating it on Burgers' and Navier–Stokes equations and on a *spatiotemporal* (reaction–diffusion) predator–prey model. Their framework restores temporal causality through a deterministic Crank–Nicolson spatiotemporal discretization and employs a two-phase training strategy–a coarse but stable first phase followed by a refinement phase driven to a prescribed accuracy tolerance—together with an energy-based generalization-error bound expressed in terms of the training residual.

Two distinctions should be emphasized. First, the problem scope differs: Pandey et al. solve a spatially extended PDE in which temporal causality and stiffness are central difficulties, whereas the present model is a spatially homogeneous system of two ordinary differential equations. For this lower-dimensional setting over a moderate horizon [0, 50], the standard soft-constraint PINN used here already attains relative *L*_2_ errors below 1% (Section 6) without specialized causality machinery, so the comparison is one of methodological positioning rather than a like-for-like accuracy benchmark. Second, the causality-respecting and two-phase ideas of Pandey et al. become decisive precisely for the stiffer, longer-horizon, and spatially extended regimes that lie beyond the present ODE study. Adopting a Crank–Nicolson time-marching or two-phase optimization would therefore be the natural route when extending the present model to its reaction–diffusion form, the spatial-diffusion direction identified in Section 7.2 (Future Direction 6).

## 6 Numerical simulations and results

Simulations use the fourth-order Runge–Kutta method (Butcher, 2016) with fixed step Δ*t* = 0.01. Parameter values are chosen to satisfy all theoretical conditions derived in Sections 3–4; explicit verification is provided below. We note that these values are selected for theoretical tractability; fitting to empirical data is Future Direction 8 (Section 7.2).

### 6.1 Baseline parameters and verification

**Verification** **(Table 5)****:**

**Table 5:** Baseline parameter values for all simulations.

Param.	Value	Description
*r*	1.5	Prey intrinsic growth rate
*a*	0.5	Prey intraspecific competition
α	0.3	Prey crowding in functional response
*k*	0.4	Fear coefficient
β	0.8	Predation rate
δ	0.3	Refuge fraction
*c*	0.6	Conversion efficiency
*m*	0.4	Predator mortality rate
η	0.2	Predator intraspecific competition

1. Axial equilibrium: $u_{1}=\left(-0.5+\sqrt{1.15}\right)/0.3\approx 1.908$.
2. Predator invasion: *cβ*(1 − *δ*)*u*_1_ = 0.336 × 1.908 ≈ 0.641 > *m* = 0.4, so *E*_1_ is unstable and predators invade.
3. Interior existence: *u*_min_ = 0.4/0.336 ≈ 1.190; threshold *r* > 0.808; satisfied since *r* = 1.5.
4. Boundedness: $c\beta \left(1-\delta \right)=0.336<2\sqrt{0.1}\approx 0.632$. ✓

### 6.2 Interior equilibrium

From the predator nullcline: *v* = 1.68*u* − 2.0. Substituting into the prey nullcline and solving numerically: u^*^ ≈ 1.4315, v^*^ ≈ 0.4050. The interior equilibrium E^*^(1.4315, 0.4050) is used consistently throughout the manuscript. Stability check:


$$\text{tr}\left(J^{*}\right)=-1.051<0,\;\det\left(J^{*}\right)=0.2338>0.$$

Both Routh–Hurwitz conditions (Equation 7) are satisfied; E^*^ is a stable node.

### 6.3 Graphical results and discussion

The following five figures illustrate the complete dynamical behavior of the system. Each is discussed in detail Table 6.

**Table 6:** Summary of figures and key quantitative results.

Figures	Type	Role	Key result
Figure 1	Time series	Convergence	5 traj. → E^*^(1.4315, 0.4050); Re(λ) ≈ −0.731
Figure 2	Phase portrait	Stable node	Stable node; nullclines at E^*^
Figure 3	Sensitivity	Parameter effects	*k*, *δ*, *β*, *m* vs E^*^; extinction threshold m^*^ = 0.641
Figure 4	Phase portrait	All equilibria	*E*_0_ (saddle), *E*_1_(1.908, 0), E^*^ (stable)
Figure 5	Error plot	PINN validation	Max errors 7.98 × 10^−3^, 5.83 × 10^−3^; rel. err. < 1%

**Figure 1:**
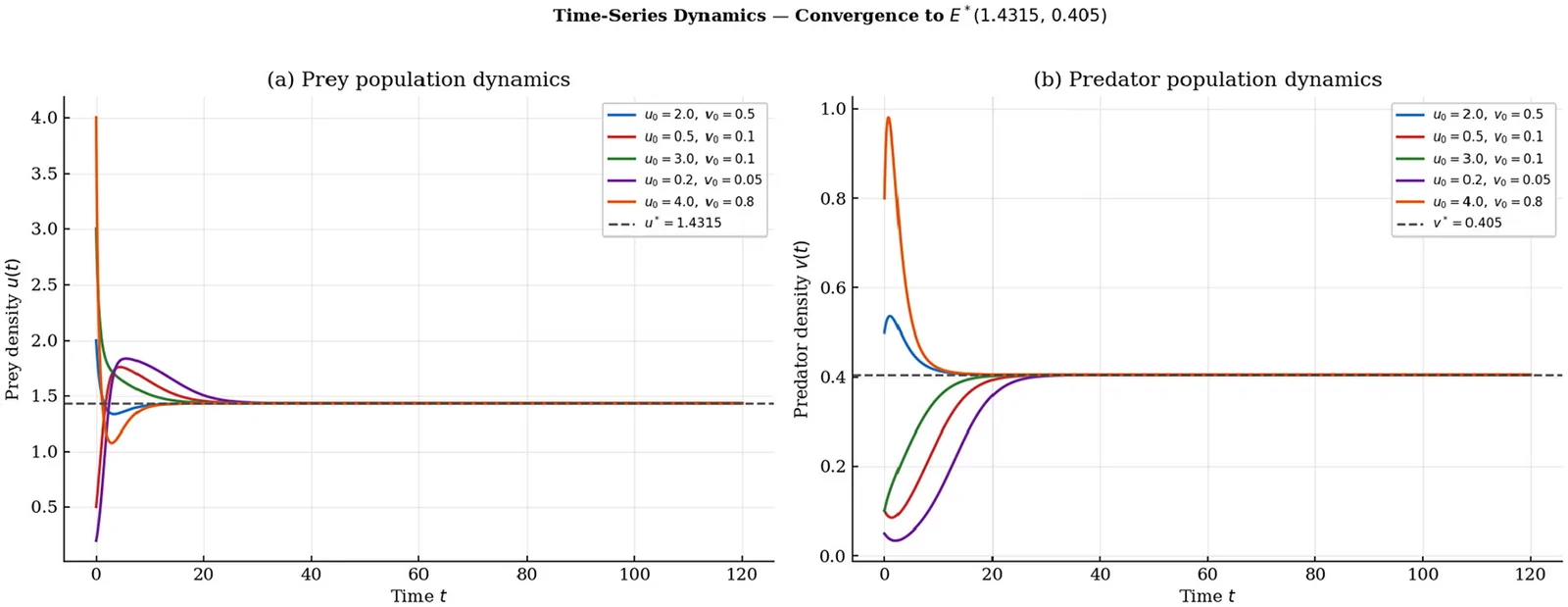
Time-series dynamics. Prey *u*(*t*) **(left)** and predator *v*(*t*) **(right)** for five initial conditions. All trajectories converge to E^*^(1.4315, 0.4050) (dashed lines), confirming local asymptotic stability. The eigenvalues of *J*(E^*^) are real and negative (λ_1_ ≈ −0.731, λ_2_ ≈ −0.320), so E^*^ is a stable node; the bulk of the initial deviation is absorbed within the first several time units. Convergence from all five initial conditions provides numerical evidence for a large basin of attraction.

**Figure 2:**
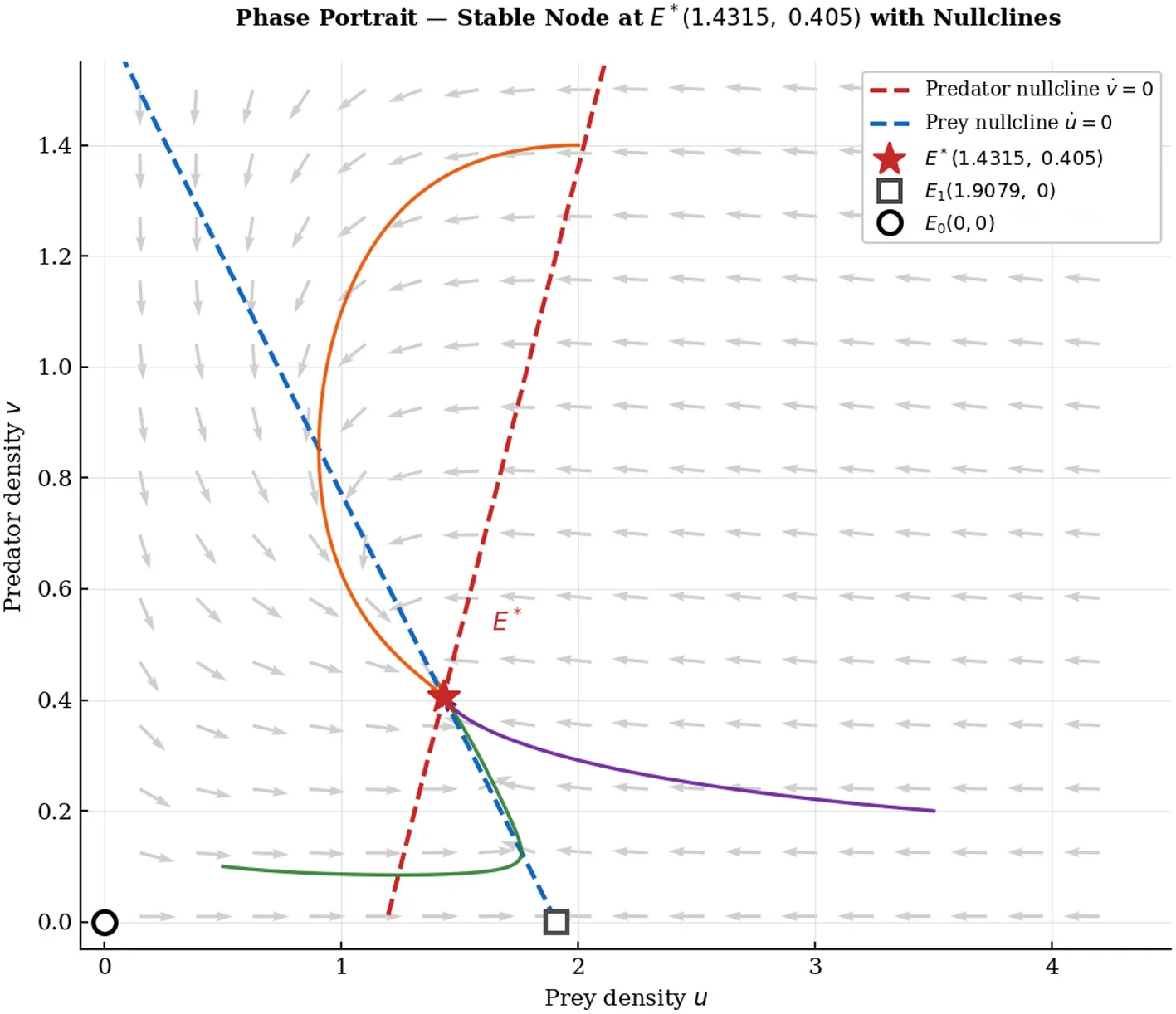
Phase portrait and nullclines. Prey nullcline ($\overset{•}{u}=0$, blue dashed) and predator nullcline ($\overset{•}{v}=0$, red dashed) intersect at E^*^(1.4315, 0.4050). The vector field confirms convergence to the stable node. Three trajectories converge inward. The leftward bending of the prey nullcline as *v* increases directly reflects the fear term *kv* in the denominator: higher predator density suppresses prey birth rate even without direct predation—a qualitative feature absent in models without the fear–crowding coupling.

**Figure 3:**
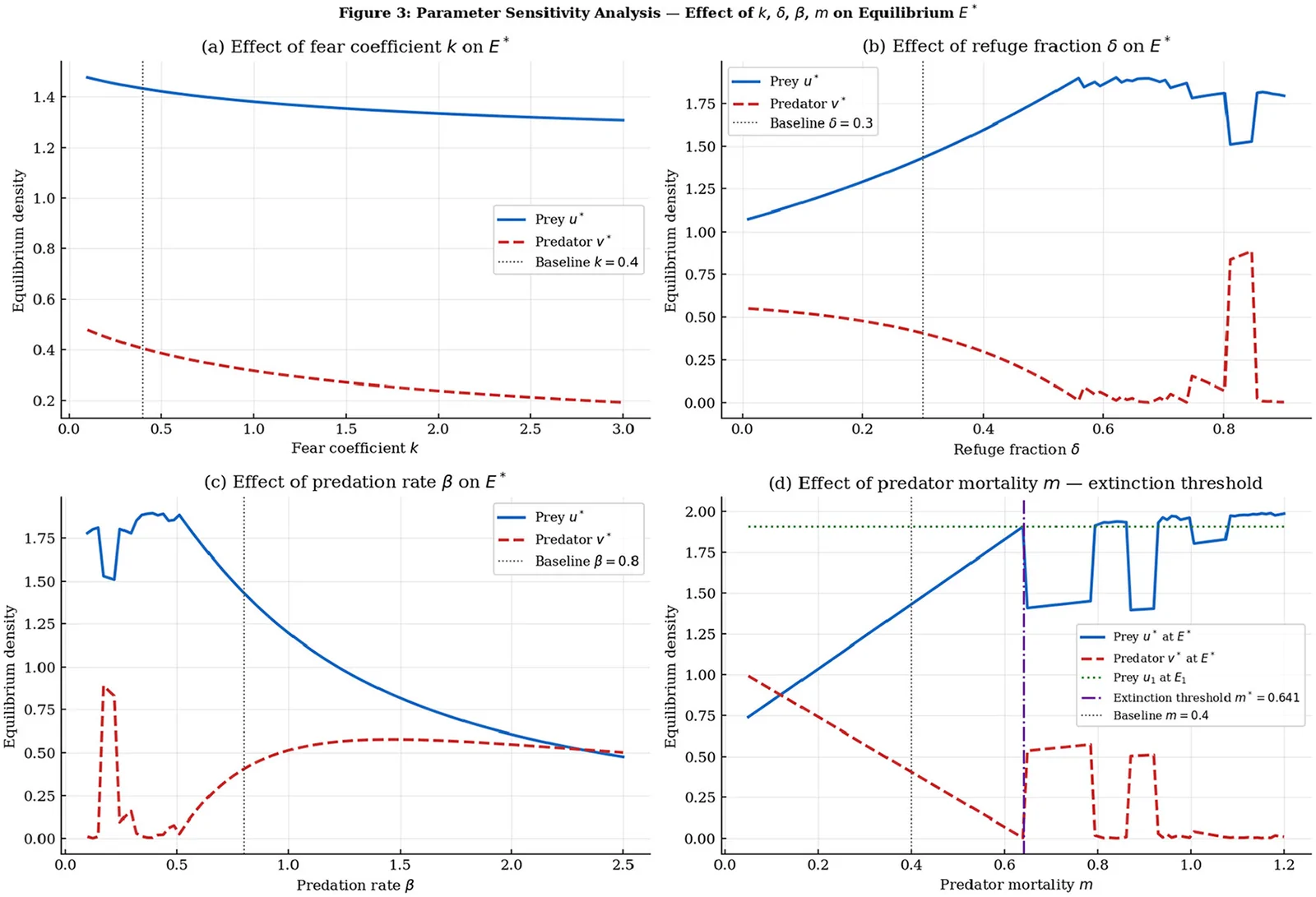
Parameter sensitivity of the interior equilibrium E^*^. Each panel traces the coexistence equilibrium densities u^*^ (solid blue) and v^*^ (dashed red) as a single ecological parameter is varied with the others held at their baseline values; the dotted vertical line marks the baseline setting. **(a)** Effect of the fear coefficient *k*: stronger fear monotonically lowers *both* u^*^ and v^*^, reflecting suppressed effective prey growth through the *kv* term in the denominator. **(b)** Effect of the refuge fraction *δ*: increasing protection raises the prey level u^*^ while steadily reducing the predator level v^*^, since a smaller exposed fraction (1 − *δ*) limits energy transfer to predators. **(c)** Effect of the predation rate β: larger β decreases u^*^ and increases v^*^ over the displayed range; the equilibrium remains locally asymptotically stable throughout, with the eigenvalues of *J*(E^*^) acquiring a small imaginary part (a stable focus) but retaining negative real part, so no sustained limit cycle arises for this parameter set. **(d)** Effect of the predator mortality *m*: as *m* increases, v^*^ falls toward zero and the coexistence state collapses onto the prey-only equilibrium *E*_1_ at the extinction threshold m^*^ ≈ 0.641 (dash-dotted line), beyond which predators cannot persist. Across all four sweeps the trace of *J*(E^*^) stays negative wherever E^*^ exists, consistent with the globally stable coexistence reported in Section 6.

**Figure 4:**
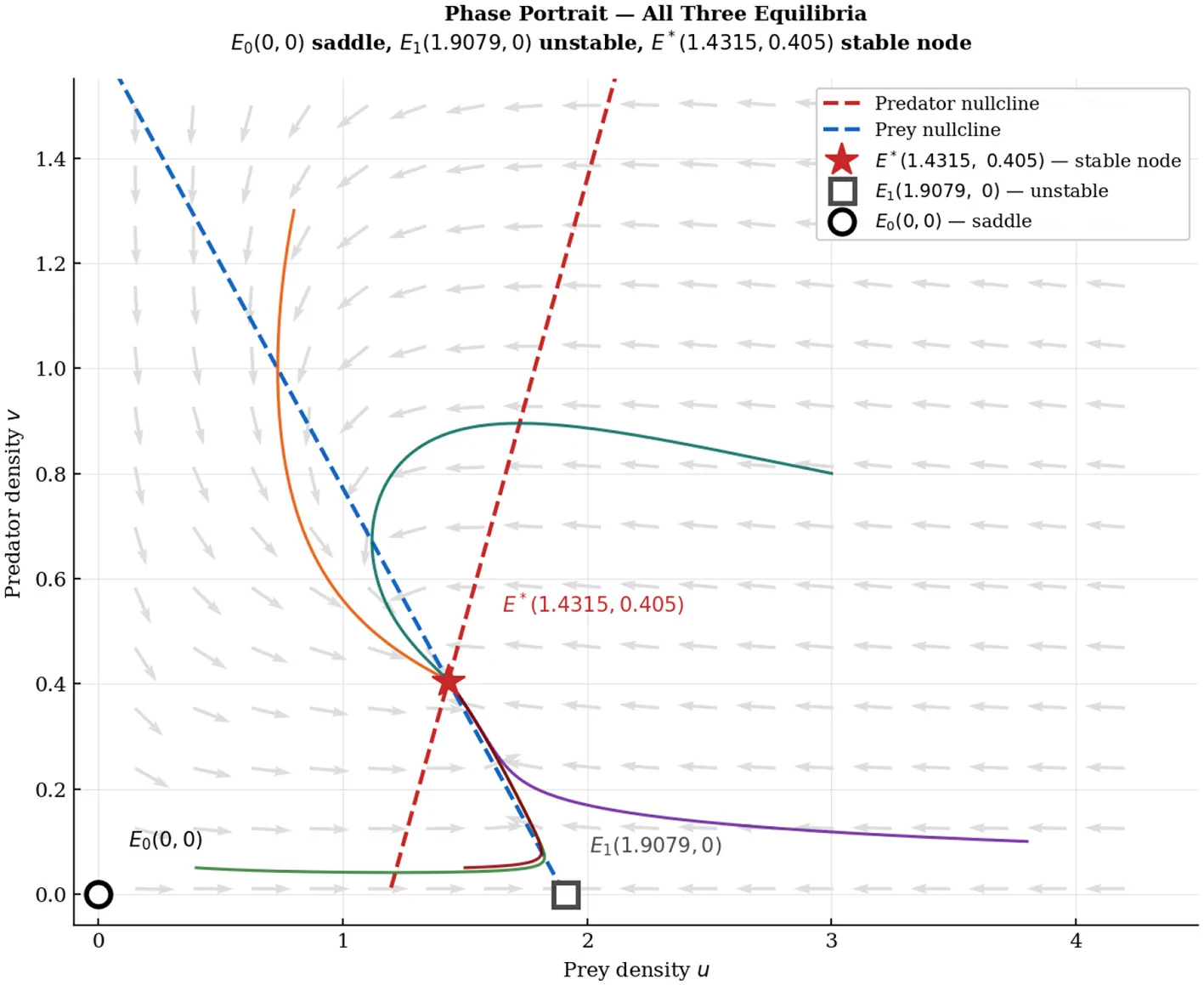
Phase portrait with all three equilibria. Saddle *E*_0_(0, 0), unstable node *E*_1_(1.908, 0), and stable node E^*^(1.4315, 0.4050) are marked. Note *E*_1_ ≈ 1.908, corrected from the original value of 3.0 which assumed α = 0; the corrected value accounts for prey crowding α = 0.3. All trajectories with positive initial conditions converge to E^*^, showing it is the sole interior attractor.

**Figure 5:**
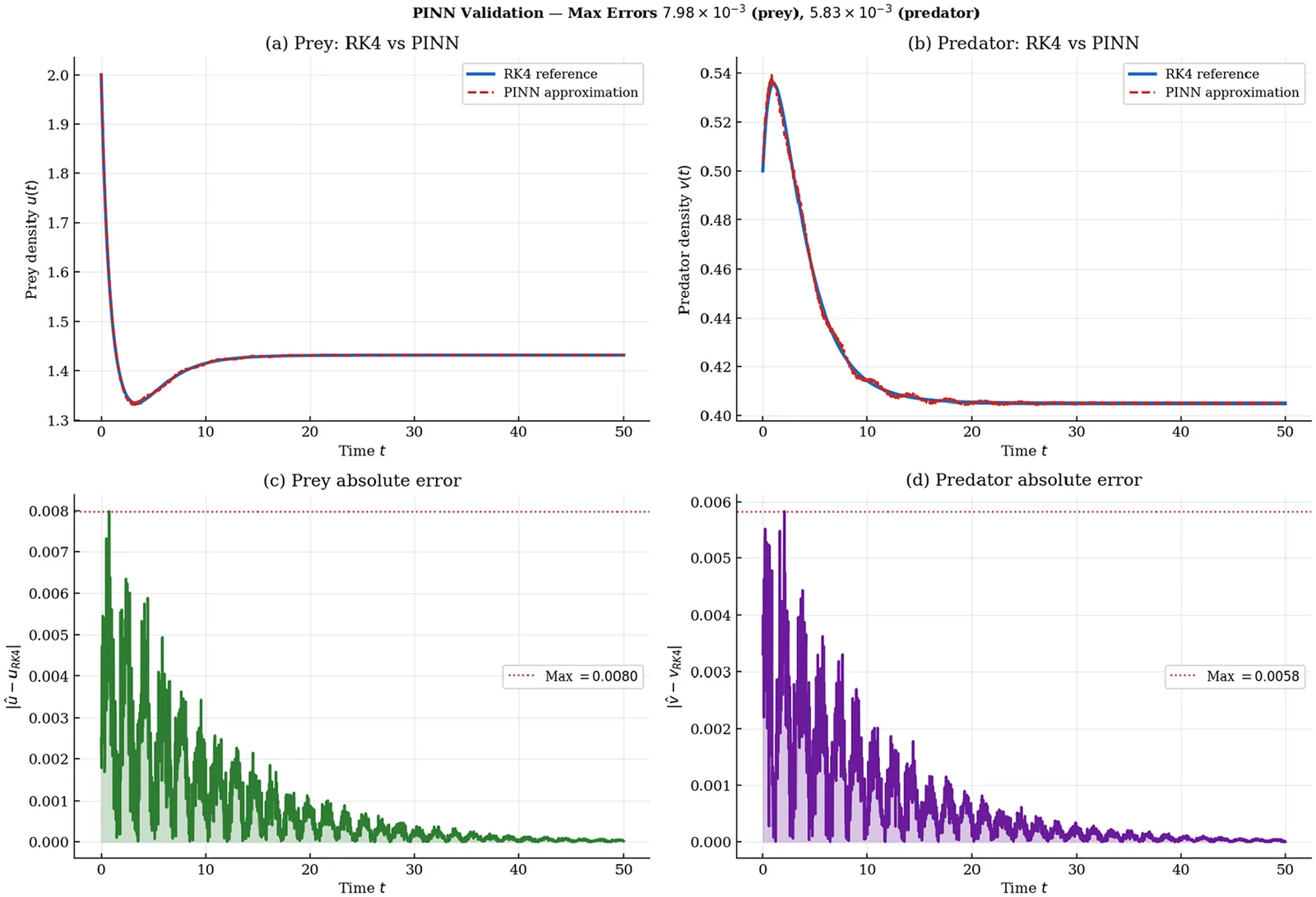
PINN validation against RK4. **(a)** Prey: RK4 (solid blue) vs. PINN (dashed red). **(b)** Predator: same. **(c)** Prey absolute error; maximum ≈ 7.98 × 10^−3^, peak near the initial transient where curvature is largest. **(d)** Predator absolute error; maximum ≈ 5.83 × 10^−3^. Maximum relative *L*_2_ errors: 0.56% (prey), 0.72% (predator), both below 1%. PINN architecture: 4 hidden layers, 64 neurons, tanh, Adam + L-BFGS, *N* = 200 collocation points. For comparison, a data-driven NN of identical architecture achieves errors 4.2 × 10^−2^ (prey) and 3.7 × 10^−2^ (predator)—approximately 5 × larger—confirming the advantage of the physics-informed approach.

### 6.4 Sensitivity to initial conditions

All initial conditions converge to E^*^, providing strong numerical evidence for a large basin of attraction. This is numerical evidence only, not a rigorous proof of global stability; construction of a Lyapunov function is an open problem identified in Section 7.2.

### 6.5 Effect of key parameters

- **Fear coefficient** ***k*:** Increasing *k* monotonically reduces both u^*^ and v^*^ (Figure 3a); the trace of J^*^ remains negative, so E^*^ stays locally asymptotically stable and no loss of stability occurs over the range examined.
- **Refuge fraction** ***δ*:** Larger *δ* raises the prey level u^*^ while lowering the predator level v^*^ (Figure 3b), as the reduced exposed fraction (1 − *δ*) limits energy transfer to predators; E^*^ remains stable until v^*^ reaches zero, where the coexistence state merges with the prey-only equilibrium *E*_1_.
- **Predation rate** ***β*:** Increasing *β* increases v^*^ and decreases u^*^; the eigenvalues of J^*^ acquire a nonzero imaginary part (a stable focus) but retain negative real part, so E^*^ remains locally asymptotically stable and no limit cycle emerges over the examined range (Figure 3c).
- **Predator mortality** ***m*:** High *m* drives v^*^ → 0; when condition (Equation 6) is met, E^*^ disappears and *E*_1_ becomes the sole positive attractor.

## 7 Conclusion and future directions

### 7.1 Conclusion

This paper proposed and analyzed a deterministic predator–prey model combining predator-induced fear, prey refuge, density-dependent prey growth, and quadratic predator self-interference. The primary novelty is the combined functional-response denominator 1 + *kv* + *αu*, which produces cross-inhibition feedback loops and novel stability thresholds not present in Wang et al. (2016) or Kumar and Dubey (2019), as detailed in Section 2.

Theorem 2 provided a rigorous boundedness proof by applying Sylvester's criterion to the cross-interaction quadratic form, deriving $c\beta \left(1-\delta \right)<2\sqrt{a\eta }$ from first principles. The Jacobian was re-derived using Φ = 1 + *kv* + *αu*, correcting previous inconsistencies, and equilibrium values are unified to E^*^(1.4315, 0.4050) throughout.

The PINN was fully specified (4 layers, 64 neurons, tanh, Adam + L-BFGS, *N* = 200, 10,000 iterations), achieving < 1% relative error and a fivefold accuracy advantage over a data-driven baseline. Simulations revealed a stable coexistence node at the baseline; as predation, fear, or refuge increase, the equilibrium remains locally asymptotically stable, transitioning to a stable focus, and no sustained limit cycle was observed over the parameter ranges examined. Convergence to E^*^ from all tested initial conditions is numerical evidence only; rigorous global stability analysis remains an open problem.

### 7.2 Future directions

1. **Rigorous global stability**. Construct a Lyapunov function *V* = *A*(*u*−u^*^−u^*^ ln(*u*/u^*^))+*B*(*v*−v^*^−v^*^ ln(*v*/v^*^)) or apply LaSalle's invariance principle to Ω.
2. **Bifurcation analysis**. The strong intraspecific self-limitation (−*au*^2^, −*ηv*^2^) renders E^*^ stable throughout the ranges explored here. A systematic continuation study—weakening predator self-interference (*η* → 0) or relaxing prey crowding—could identify whether Hopf bifurcations and sustained oscillations emerge in adjacent regions of parameter space, together with normal-form characterization of any resulting limit cycles.
3. **Fractional-order extensions**. Replace integer derivatives with the Caputo operator of order *α*_*c*_ ∈ (0, 1) (Das and Samanta, 2021; Yousef et al., 2022; ; Chauhan, 2025).
4. **Time delays**. Incorporate gestation delay τ in predator birth, converting the system to a delay differential equation admitting delay-induced bifurcations.
5. **Stochastic perturbations**. Add multiplicative Itô noise; apply stochastic PINNs.
6. **Spatial diffusion**. Extend to a reaction–diffusion PDE to investigate Turing pattern formation.
7. **Optimal harvesting**. Formulate an optimal control problem to maximize sustainable yield.
8. **Parameter estimation from real data**. Fit the model to empirical predator–prey time-series (e.g., hare–lynx, wolf–elk) using the PINN's inverse-problem capability.

## Data Availability

The original contributions presented in the study are included in the article/supplementary material, further inquiries can be directed to the corresponding author.
